# Supplementary material for: Outcomes of early NIH-funded investigators: Experience of the National Institute of Allergy and Infectious Diseases
Source: PLoS One. 2018 Sep 12;13(9):e0199648. doi: 10.1371/journal.pone.0199648 (PMC6135352; doi:10.1371/journal.pone.0199648)
Supplement: S1 Appendix — (DOCX) [file pone.0199648.s001.docx]

**S1. Appendix**

**Details of cohort selection and identification of project start dates in IMPAC II**

From the original data set of 25,125 awards extracted from IMPAC II, we first had to identify which awards were made to “first-time” R01-e awardees versus those awards made to established investigators.

Prior to receiving the first R01 award, a PI is considered a “New Investigator” (NI), defined as an investigator who has not previously received substantial, independent funding from NIH [1]. Once a NI receives their first R01 award, the FY of that award becomes the investigator’s official first R01 award year, and the PI’s status changes from NI to EI.

Two data fields in IMPAC II were used to identify the first-time R01 awardee. The first was a NI flag on the application identifying the PI’s status as NI. The second was a data field called “PI first R01 award FY” (FRAY) which contains the FY of the PI’s first R01 award. If the FRAY equaled the FY of that R01 award, that allowed us to identify a first-time award and awardee. R01-e awards other than R01 were not marked as NI because they were not considered R01-e awards by NIH at the time, but if the FRAY data field was empty, that identified a first-time award and awardee. In either case, if the FRAY data field was not empty, and the date was earlier than the FY of current award, that identified an EI and ruled out that investigator as a first-time awardee.

Nevertheless, we were aware that NI status information on R01 applications and FRAY data in IMPAC II, were inherently inaccurate in some cases and could not be used alone to select the cohort. Prior to 2010, NI status was self-reported by PI on R01 applications. Consequently, FRAY data were often missing or inaccurate. It was not uncommon, for example, to find R01 awards where the application was marked “NI” and the corresponding FRAY was equal to the FY of that award, when in fact the PI had received an R01 award years earlier. The errors were compounded as the NIH funding environment became more competitive and preferential NI paylines were introduced in 2006 [2]. It was not until 2010, when all grant applications had to be submitted on electronic forms, and the PI biographical sketch was one of those, did these errors drastically decrease [3].

To work around the poor quality of application NI status and FRAY data in IMPAC II, we took a stepwise approach to identifying unique PI and ordering all their applications chronologically. We sorted the data in the following hierarchy: PI profile ID; application FY; serial number; project number; application ID. Second, we created a new study-specific data field called the Index Fiscal Year (or IFY, as referred to above). We used the following algorithm to populate this field: If the award FY (AFY) was less than the FRAY , then the IFY was set to equal the AFY. If the AFY equaled the FRAY, then the IFY was set to equal the FRAY. If the AFY was greater than the FRAY, then the IFY was set to equal the FRAY. If the FRAY was missing (indicating the award was an R01-e *other than an R01*), then the IFY was set to the AFY. (Algorithm “if/then” statements were: if AFY < FRAY, then IFY = AFY; If AFY = FRAY, then IFY = FRAY; If AFY > FRAY, then IFY = FRAY; If FRAY = n/a, then IFY = AFY)

From the list of awardees there were 3 groups: 1) those who received their first R01-awards from NIAID; 2) those who received an R01-e award other than R01 from NIAID and had not received an earlier R01 award from any NIH Institute (IC); and 3) those who received R01-e awards other than R01 from NIAID and an earlier R01 award from another IC. We excluded the third group, because we wanted to focus on investigators who received their first awards from NIAID. In total, we identified 1,496 investigators who received their first R01-e awards from NIAID between 2003 and 2010. We called the cohort investigators “Early NIH-funded Investigators” (ENI).

In order to study the grant submission behavior of the cohort, we took the PI profile IDs of the 1,496 ENI and searched the IMPAC II database for all R01-e grant applications they submitted to any IC between 2003 and 2016. We restricted the search to “contact PI” only, which prevented extracting duplicate records of multiple PI applications, because the NIH multiple PI policy was not in place until FY2007 [4]. The search extracted 12,964 applications.

From the data set of 12,964 applications, we had to: 1) identify ENI index awards; 2) chronologically order applications for each ENI; and 3) calculate the time between the index award and subsequent applications for each ENI. Thus, a critical application-associated data field was the project start date.

Using the “project start date” field in IMPAC II was complicated, however, by the following issues: 1) a number of renewal projects had project start dates earlier than the ENI’s IFY; these were projects begun under a different PI and passed on to the ENI who submitted the next competitive segment; 2) subproject awards did not have project start dates; 3) project start dates (especially those before 2010) were often erroneous, for reasons that are unclear. These complications became obvious when we began to calculate the time between the index award and subsequent application submissions.

Although most applications had accurate project start dates, there were so many cases of erroneous dates we chose to use the National Advisory Allergy and Infectious Diseases (NAAAID) Council Date as a proxy for project start date. We took the NIAID average time from NAAID Council dates to notice-of-award date – i.e. 4 months – and added this to the application Council Date to derive an estimated project start date. We applied this approach uniformly to all applications (except 57, which were missing Council dates but had accurate project start dates).

**S1 Appendix references**

1. National Institutes of Health, Office of Extramural Research. Early Stage and Early Established Investigator Policies. Available from: <https://grants.nih.gov/policy/early-investigators/index.htm>
2. National Institutes of Health, Office of Extramural Research. A History of New and Early Stage Investigator Policies and Data. Available from: <https://grants.nih.gov/policy/early-investigators/history.htm>
3. National Institutes of Health, Office of Extramural Research. Enhancing Peer Review. Available from: <https://enhancing-peer-review.nih.gov/faqs.html#2993>
4. National Institutes of Health, Office of Extramural Research. Establishment of Multiple Principal Investigator Awards for the Support of Team Science Projects, Notice Number: NOT-OD-07-017. Available from: <https://grants.nih.gov/grants/guide/notice-files/NOT-OD-07-017.html>
