# Supplementary material for: Outcomes of early NIH-funded investigators: Experience of the National Institute of Allergy and Infectious Diseases
Source: PLoS One. 2018 Sep 12;13(9):e0199648. doi: 10.1371/journal.pone.0199648 (PMC6135352; doi:10.1371/journal.pone.0199648)
Supplement: S1 Table — (DOCX) [file pone.0199648.s002.docx]

**S1 Table. Mean Number of Years ENI Continued to Apply for NIH Grants**

| **Cohort Year** | **Unfunded ENI** | **Funded ENI** | **Difference** | **All ENI** |
| --- | --- | --- | --- | --- |
| 2003 | 4.86 | 11.18 | 6.3 | 9.07 |
| 2004 | 5.95 | 10.68 | 4.7 | 8.82 |
| 2005 | 6.03 | 9.67 | 3.6 | 8.82 |
| 2006 | 4.67 | 8.95 | 4.3 | 7.23 |
| 2007 | 4.91 | 7.93 | 3.0 | 6.47 |
| 2008 | 5.08 | 7.08 | 2.0 | 6.21 |
| 2009 | 5.09 | 6.26 | 1.2 | 5.68 |
| 2010 | 3.88 | 5.35 | 1.5 | 4.70 |
| **All Cohort Years** | **5.06 (median 5.42)** | **8.39 (median 9.04)** | **4.00 (median 3.62)** | **7.12 (median 8.23)** |
| Based on 1496 ENI; table shows mean number of years per cohort year. | | | | |
