# Supplementary material for: Outcomes of early NIH-funded investigators: Experience of the National Institute of Allergy and Infectious Diseases
Source: PLoS One. 2018 Sep 12;13(9):e0199648. doi: 10.1371/journal.pone.0199648 (PMC6135352; doi:10.1371/journal.pone.0199648)
Supplement: S2 Table — (DOCX) [file pone.0199648.s003.docx]

**S2 Table. ENI Funding Success According to Index Award Score Above or Below NI Payline**

| **Index Award** | **# ENI Funded** | **# ENI Unfunded** | **Total # ENI** | **% ENI Successful^*^** |
| --- | --- | --- | --- | --- |
| **Paid <= PL** | 274 | 183 | 457 | **60%** |
| **Paid > PL** | 91 | 100 | 191 | **48%** |
| ^*^Pearson's χ^2^ test, *p*-value < 0.004 | | | | |
