# Supplementary figures and images for: Outcomes of early NIH-funded investigators: Experience of the National Institute of Allergy and Infectious Diseases
Source: PLoS One. 2018 Sep 12;13(9):e0199648. doi: 10.1371/journal.pone.0199648 (PMC6135352; doi:10.1371/journal.pone.0199648)

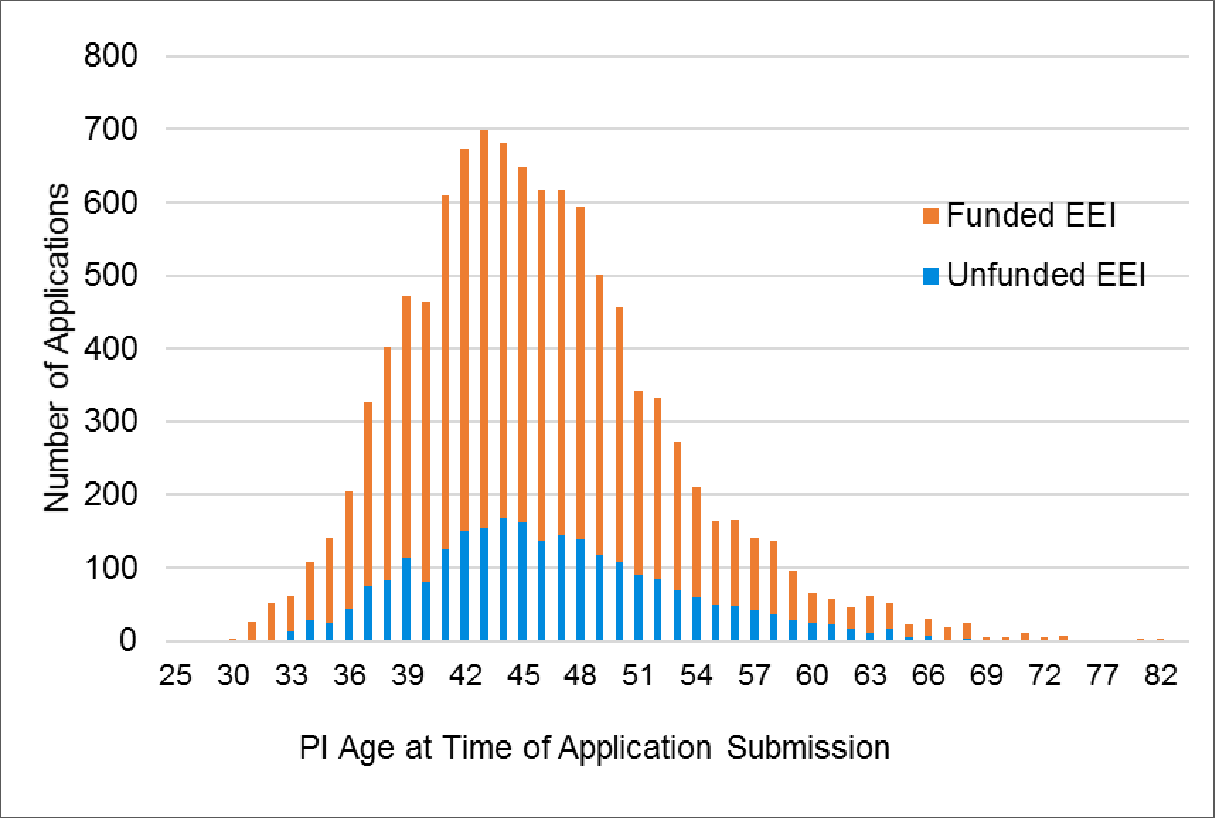

Supplement: S1 Fig — (TIF) [file pone.0199648.s004.tif]

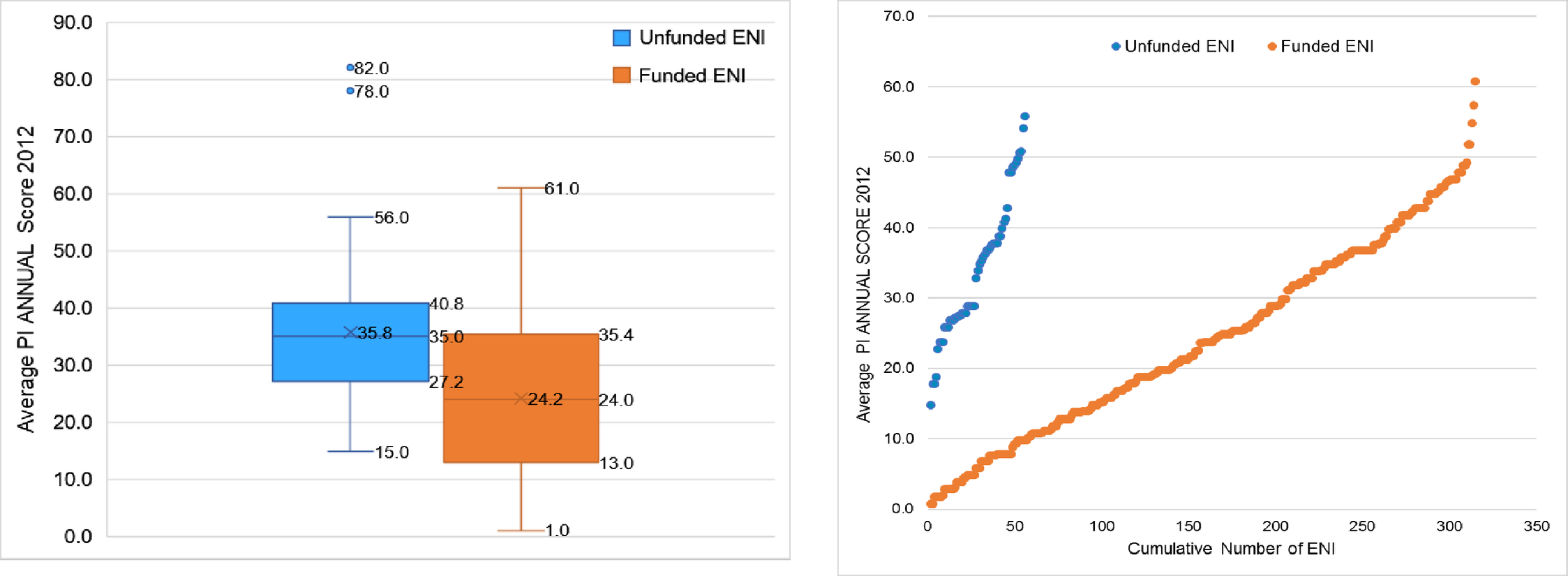

Supplement: S2 Fig — a) ENI Average Application ANNUAL Scores, FY12, b) ENI Average Application ANNUAL Scores and cumulative numbers of ENI, FY12. (TIF) [file pone.0199648.s005.tif]

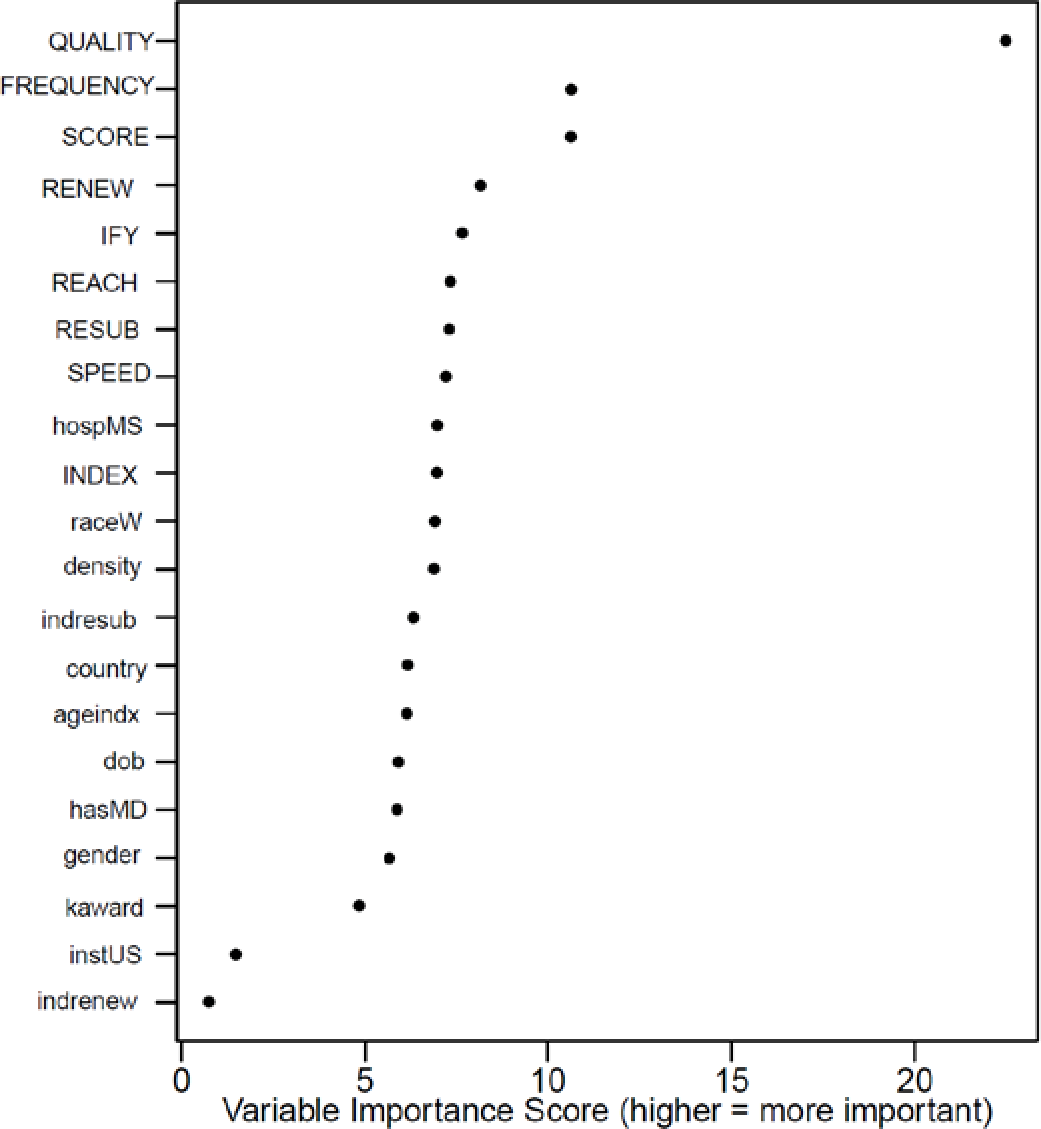

Supplement: S3 Fig — (TIF) [file pone.0199648.s006.tif]
